# Investigation of Wind Field Characteristics in Mountain Valley Terrain Under the Disturbance of Bridge Structures

**DOI:** 10.3390/s26072098

**Published:** 2026-03-27

**Authors:** Chaoming Wu, Junrui Zhang, Hongbo Yang, Hao Liu, Rujin Ma

**Affiliations:** 1 Guizhou Bridge Construction Group Co., Ltd., Guiyang 550001, China; 13765178260@163.com (C.W.); howe_ty@163.com (H.L.); 2 College of Civil Engineering, Tongji University, Shanghai 200092, China; rjma@tongji.edu.cn; 3 Guizhou Transportation Planning Survey & Design Academe Co., Ltd., Guiyang 550081, China; yanghongbo111@sina.cn

**Keywords:** mountain valleys terrain, wind field characteristics, long-span bridge, structure disturbance effect

## Abstract

This study investigates the wind field characteristics of long-span suspension bridges in mountain valleys terrain, with a particular focus on the disturbance effects caused by bridge structure on wind measurements. Field data are collected using the Wind3D 6000 LiDAR installed near the bridge. By comparing wind field characteristics before and after bridge completion, this study evaluates the influence of the bridge structure on both mean and turbulent wind characteristics. The findings show that the presence of the bridge tower and deck reduces the measured mean wind speed and modifies its probability distribution. The bridge tower increases the effective ground roughness coefficient, thereby attenuating the vertical wind speed gradient. In addition, the bridge tower raises the measured turbulence intensity, alters its probability distribution, and decreases the agreement between the turbulent wind power spectrum and the von Kármán spectrum. It is necessary to correct the data affected by these disturbances to improve the accuracy of wind load assessments for long-span bridges, thus enhancing the reliability of bridge structural operation.

## 1. Introduction

Long-span bridges, due to their low stiffness and damping characteristics, are particularly vulnerable to wind loads and susceptible to wind-induced vibrations [1,2,3]. Furthermore, the wind field in mountain valley regions is highly complex and variable, with significant fluctuations in wind speed influenced by the topography of the terrain [4,5,6]. As a result, ensuring the structural safety and operational reliability of bridges in such dynamic wind environments is crucial in their design and management [7]. The primary objective of this study is to thoroughly understand the wind field characteristics in complex terrain, including both mean wind characteristics and turbulent wind behavior [8,9], in order to support the wind-resistant design of bridge structures.

The field measurement method directly captures wind parameters, such as wind speed and direction, thereby providing an accurate representation of wind-field characteristics. As a result, it has become the most effective approach for studying wind field characteristics in mountain valleys terrain. At the same time, Coherent Doppler LiDAR has shown exceptional performance in wind field measurements and has become widely used by researchers [6,10,11,12]. In particular, in the complex terrain of mountain valleys, LiDAR-based wind measurement systems enable comprehensive data collection through three-dimensional wind field scanning. Furthermore, the data captured by LiDAR can be stored locally and uploaded to the cloud, facilitating real-time updates [13]. This capability provides a robust foundation for long-term monitoring and analysis of wind field characteristics.

Building on field measurement data, many researchers have investigated wind field characteristics in valleys. Numerous studies have shown that the characteristics of the mountain valleys wind field differ from the national design standards [14,15] or codes. These differences in parameters include mean wind speed [16,17], turbulence intensity [18], mean wind profile shapes [19], surface roughness values [4], and power spectrum models [6]. Some studies have examined the unique effects of mountain valley wind fields, including terrain-induced acceleration of mean wind speed [20], and the alignment of the prevailing wind direction with the valley’s orientation due to topographical constraints [17,21]. Wind speed exhibits features of monsoonal circulation, with distinct diurnal variations [22,23]. Additionally, several studies have developed probabilistic models for wind field parameters based on field data [5,24], exploring the relationships between these parameters [25]. Field measurement data provide critical insights into the aerodynamic environment at bridge sites in mountainous valleys, thereby supporting design validation and operational safety assessment.

To achieve accurate characteristics of complex wind field in mountain valleys terrain, the acquisition of high-quality wind data is essential. However, following bridge construction, interactions between the structure and the surrounding wind environment may introduce disturbances into the measured wind data, leading to anomalous observations. For instance, long-term on-site wind monitoring at the Sutong Bridge has revealed abnormal distributions of turbulence intensity [26,27]. In addition, the inherent flexibility of long-span bridges leads to complex structural vibrations, which can couple with aerodynamic disturbances and further compromise the accurate measurement of the ambient wind field [28]. Such structural interference significantly affects both mean wind speed and turbulence intensity and exhibits a strong dependence on wind direction [29]. It can also influence the probabilistic modeling of wind field parameters [30]. Consequently, these effects may cause misinterpretation of wind field characteristics and lead to inaccuracies in wind load assessment.

Therefore, before wind field analysis, the interference effects must be systematically examined and the measured wind data appropriately corrected. Ma et al. [29] performed a comprehensive study on the interference effects of bridge deck on wind field measurements and provided mechanistic interpretations of these phenomena. By introducing disturbance coefficients to quantitatively characterize structural interference, they developed a statistical model to correct wind field data affected by such disturbances. This approach was successfully applied to wind data correction for long-span cable-stayed bridges in coastal regions of China, demonstrating strong effectiveness. Building on the methodology proposed by Ma et al., Zuo et al. [31] further established a wind data correction model for the Runyang Bridge and verified its applicability under the wind field conditions of long-span suspension bridges. In contrast, Chen et al. [32] found that interference from bridge deck structures can lead to anomalous wind data distributions, and, based on this observation, proposed a deep learning–based data-cleaning framework to reconstruct the ambient wind field from disturbed measurements. Their method enabled efficient correction of wind speed data at the second level. However, the applicability of this approach remains largely confined to cable-stayed bridges in coastal environments.

Although significant progress has been made in studying the disturbed effects of bridge structures on wind field measurements, most existing research has focused on coastal regions, and studies in complex terrains, particularly mountain valleys, remain limited. With the economic development of mountainous areas, the number of bridges being constructed is increasing [33], and the operational safety of long-span bridges increasingly depends on real-time wind measurements. Therefore, investigating the interference effects of bridge structures on wind field measurements in mountain valleys terrain is of critical importance. The wind field in such regions is highly complex and variable [34,35], and the disturbed effects can differ depending on wind direction, measurement height, and location along the bridge axis. Conducting a systematic study of these effects in this unique wind environment can help correct measured data and provide a reliable basis for the safe operation of bridges.

This study take the Huajiang Valley Bridge as a case study to investigate the disturbed effects of bridge structures on the wind field in mountain valleys terrain, including both mean wind characteristics and turbulent wind behavior. This study clarifies the mechanisms of structural disturbances in a unique wind environment and provides a preliminary quantification of the interference effects, which is of significant importance for correcting sensor data and ensuring the structural safety of long-span bridges in mountainous regions. Section 2 introduces the method for obtaining wind field data and derives the formulas for wind field parameters. Section 3 analyzes the interference effects of the bridge tower and deck on the mean wind characteristics. Section 4 examines the interference effects of the bridge tower structure on turbulent wind characteristics. Section 5 concludes the study and provides final recommendations.

## 2. Engineering Background for Wind Data Collection

Huajiang Valley Bridge is located in Guizhou Province, China, connecting Anshun City with the Qianxinan Autonomous Prefecture. This bridge is a single-span double-tower steel truss suspension bridge with a total length of 2890 m, a main span of 1420 m, and a height of 625 m above the Beipan River. The bridge holds the record for the longest main span of any bridge in mountain regions and is also the highest bridge in the world. Its long span and height make it highly sensitive to wind loads. The bridge spans the Beipan River and is located above the Huajiang Valley, where the mountain valleys terrain creates a complex and variable wind environment. Therefore, monitoring the wind field is crucial for both the construction and operational safety of the structure.

**Figure 1:**
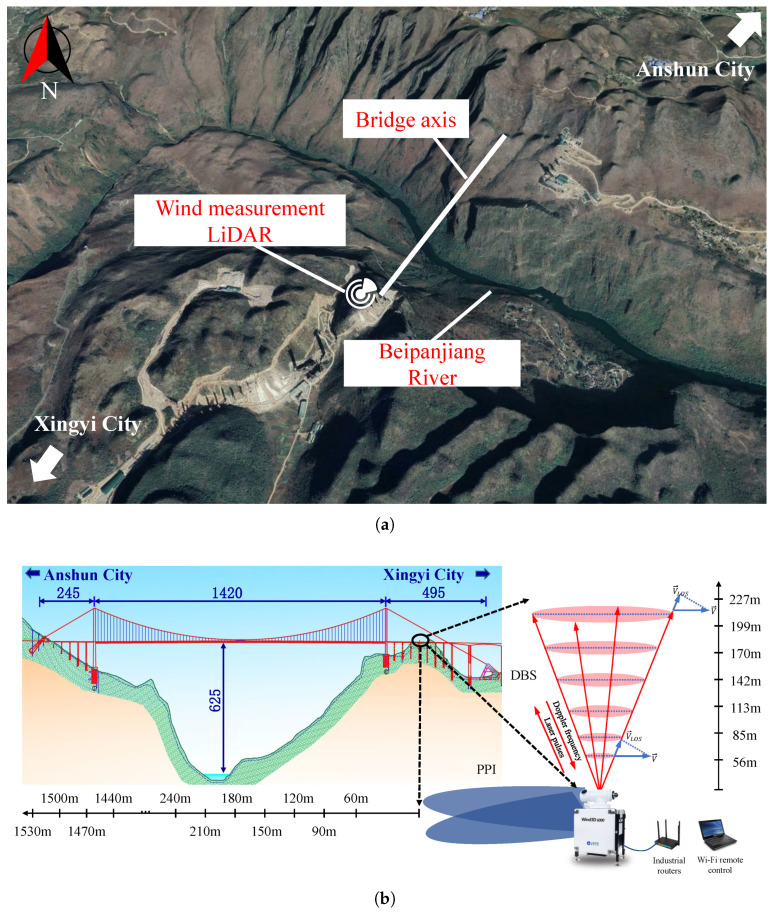
Layout of the bridge and LiDAR location. (**a**) Plane view. (**b**) Elevation view.

To monitor the wind field characteristics at the bridge site, a Wind3D 6000 LiDAR wind measurement scanning system was deployed, which enables precise detection of the 3D wind field. The accuracy of wind speed measurement is within 0.1 m/s, with a range of 0–75 m/s; the wind direction ranges from $0^{\circ }$ to $360^{\circ }$, with a resolution of $3^{\circ }$; the sampling frequency is 1.0 Hz. The LiDAR setup is shown in Figure 1a, located on a high ground to the northwest of one of the bridge’s towers. This location is 40 m from the nearest bridge tower along the bridge’s axial direction and 80 m from the nearest bridge tower in the perpendicular direction, with an elevation of 1106 m, which is nearly identical to the bridge elevation (1110 m). A horizontal line passing through the LiDAR position parallel to the bridge axial is selected as one line for measurement. At the same time, a measurement line is also arranged in the vertical direction, as shown in Figure 1b.

**Figure 2:**
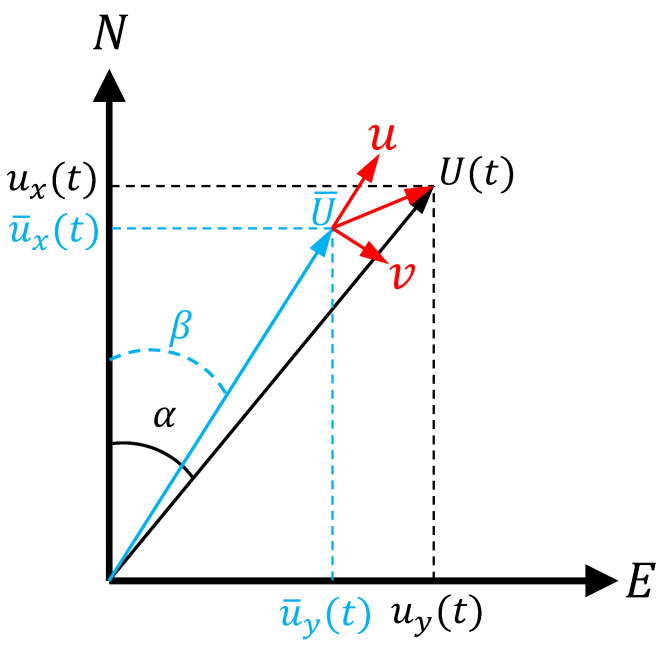
Schematic diagram of coordinate transformation during data preprocessing.

The acquired raw data are first subjected to outlier removal. Due to the low persistence of the wind direction, we employ the vector-based approach for analyzing the characteristics of wind speed [36], which incorporates both wind magnitude and direction, and offers a more accurate representation of the actual wind field than the scalar average. Figure 2 illustrates the coordinate system for vector decomposition of the data. The raw wind speed information measured by the LiDAR scanner includes the radial wind speed U(t) in the horizontal plane, wind direction α which is measured clockwise, with $0^{\circ }$ representing the true north, and the wind speed components along the *x* ($\alpha =0^{\circ }$) and *y* axes ($\alpha =90^{\circ }$) in the instrument’s coordinate system, denoted as $u_{x}\left(t\right)$ and $u_{y}\left(t\right)$, respectively. The calculation formula is as follows:

(1) $$u_{x}\left(t\right)=U\left(t\right)\cos\alpha$$

(2) $$u_{y}\left(t\right)=U\left(t\right)\sin\alpha$$

Then, the magnitude of the time-averaged wind speed ($\bar{U}$) and its corresponding direction (β) are calculated using vector algebra:

(3) $$\bar{U}=\sqrt{{\bar{u}}_{x}^{2}\left(t\right)+{\bar{u}}_{y}^{2}\left(t\right)}$$

(4) $$\beta =\arctan\left(\frac{{\bar{u}}_{y}}{{\bar{u}}_{x}}\right)$$

Following standard practice, this study uses a a 10 min averaging window as the basic time frame to calculate the average wind speed and turbulence intensity [37,38]. Unless otherwise specified, the average wind speed is calculated using a 10 min averaging window. When the number of data points within a 10 min window is fewer than 60, the sample is discarded. Taking the direction of the 10 min mean wind speed as the prevailing wind direction, the longitudinal turbulent wind *u* along the main wind direction and the lateral turbulent wind *v* are calculated using the following formulas:

(5) $$u=u_{x}\left(t\right)\cos\beta +u_{y}\left(t\right)\sin\beta -\bar{U}$$

(6) $$v=-u_{x}\left(t\right)\sin\beta +u_{y}\left(t\right)\cos\beta$$

Wind flow variability is quantified by turbulence intensity, which is defined as the normalized standard deviation (SD) of the velocity fluctuations relative to the average wind speed:

(7) $$I_{u}=\frac{\sigma_{u}}{\bar{U}}$$

(8) $$I_{v}=\frac{\sigma_{v}}{\bar{U}}$$

where $\sigma_{u}$ and $\sigma_{v}$ denote the standard deviations of *u* and *v* components. $I_{u}$ is longitudinal turbulence intensity and $I_{v}$ is lateral turbulence intensity.

To investigate the impact of the bridge structure on the wind field, data collected before (November 2024) and after (November 2025) the bridge’s completion are selected, referred to as “PC” (pre-construction) and “PoC” (post-construction). In addition, a control dataset from 26 October 2023 to 24 November 2023 is defined as “BC” (before construction) to demonstrate that the variation in the wind field is induced by the bridge structure. There are seven vertical measurement points, as shown in Figure 1b. The horizontal measurement points are represented by the distance to the bridge tower nearest to the LiDAR (referred to as “length”). Nine measurement points are chosen: 20 m, 200 m, 380 m, 560 m, 710 m (mid-span location), 860 m, 1040 m, 1220 m, and 1400 m. This study assumes that the macro-scale climatic wind characteristics of the Huajiang Valley remained statistically stationary during both the pre- and post-construction observation periods and that the wind field satisfies the quasi-steady assumption within each 10 min sampling interval. All measurements are collected using the same LiDAR through long-term continuous monitoring. The comparative analysis is carried out with consistent measurement locations and standardized data processing methods to ensure the reliability and comparability of the results. The wind field is assumed to be marginally influenced by interannual climatic variability and other natural factors, with the overall climatic background remaining stable throughout the relatively short observation period.

## 3. Effect of Bridge Structure on Mean Wind Characteristics

### 3.1. Wind Characteristics Along the Bridge Tower Axis

**Figure 3:**
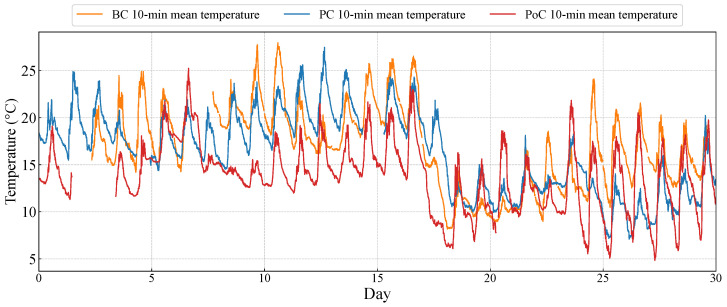
Temperature series comparison among BC, PC, and PoC periods.

To ensure the comparability of the datasets and to mitigate the influence of inter-annual meteorological variations, several control measures are implemented. Considering that seasonal synoptic patterns and thermal effects significantly influence wind characteristics in complex mountain terrains [39,40], we selected data from the similar calendar months across different years. This seasonal matching strategy ensures that the background climatic conditions and solar radiation intensity remain as consistent as possible. As shown in Figure 3, the temperature time series for both periods exhibit high consistency in both absolute values and trends, particularly the sharp decline observed after day. This suggests that the thermal forcing mechanisms driving the local valley winds are similar. By controlling for these primary environmental variables, the influence of natural climatic fluctuations is minimized.

**Figure 4:**
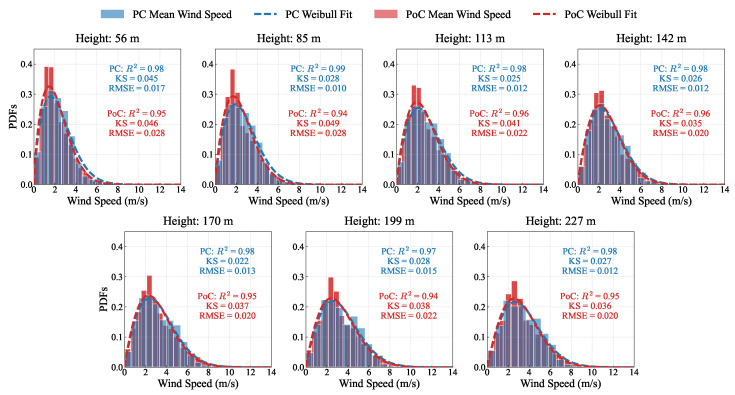
Probability density comparison of 10 min mean wind speed between PC and PoC periods at different measurement points along the bridge tower direction.

To investigate the differences in the statistical characteristics of wind speed before and after the bridge’s construction, the probability density function diagram is shown in Figure 4. It can be observed that, after the bridge’s completion, the distribution of 10 min mean wind speed becomes more concentrated, with a smaller, more defined peak. Before the bridge’s completion, as height increases, the 10 min mean wind speed gradually show a bimodal distribution with two peaks. However, this pattern is not observed after the bridge is completed. For 10 min mean wind speed, we fit the data to a Weibull distribution, which has been widely validated for modeling wind speed data due to its flexibility in capturing the characteristic shape of wind speed distributions across different meteorological conditions [41]. The probability density function (PDF) of the Weibull distribution is given by

(9) $$f\left(s;\xi \right)=\xi s^{\xi -1}\exp\left(-s^{\xi }\right)$$

where $s=\frac{x-\mu }{\sigma }$, μ is the location parameter, σ is the scale parameter, and ξ is the shape parameter.

To measure the fit quality, three statistical indicators are employed, including the Kolmogorov–Smirnov (KS) test, the coefficient of determination ($R^{2}$), and the root mean square error (RMSE). The Kolmogorov–Smirnov (KS) test is used to quantify the maximum absolute difference between the empirical cumulative distribution function (ECDF) and the theoretical cumulative distribution function (CDF). It is defined as

(10) $$D=\max|F_{\mathrm{emp}}\left(x\right)-F_{\mathrm{theo}}\left(x\right)|$$

where $F_{\mathrm{emp}}\left(x\right)$ and $F_{\mathrm{theo}}\left(x\right)$ denote the empirical and theoretical cumulative distribution functions, respectively. A smaller KS statistic indicates better agreement between the fitted distribution and the observed data. The coefficient of determination ($R^{2}$) [42] evaluates the proportion of variance in the observed data explained by the fitted model, and is expressed as

(11) $$R^{2}=1-\frac{\sum_{i=1}^{N}{\left(y_{i}-{\hat{y}}_{i}\right)}^{2}}{\sum_{i=1}^{N}{\left(y_{i}-\bar{y}\right)}^{2}},$$

where $\bar{y}$ is the mean value of the observations $y_{i}$, ${\hat{y}}_{i}$ is the fitted values. The root mean square error (RMSE) measures the average magnitude of the fitting error and is given by

(12) $$RMSE=\sqrt{\frac{1}{n}\sum_{i=1}^{n}{\left(y_{i}-{\hat{y}}_{i}\right)}^{2}}$$

**Figure A1:**
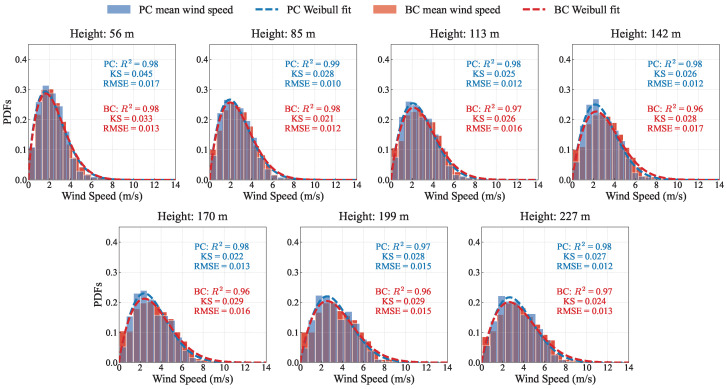
Probability density comparison of 10 min mean wind speed between BC and PC periods at different measurement points along the bridge tower.

After the bridge was constructed, the coefficient of determination ($R_{\mathrm{PoC}}^{2}$) for fitting the mean wind speed distribution at various heights using the Weibull distribution is lower than the coefficient of determination ($R_{\mathrm{PC}}^{2}$) before construction. While $R_{\mathrm{PC}}^{2}$ ranged from 0.97 to 0.99, $R_{\mathrm{PoC}}^{2}$ reached a maximum of only 0.96. Meanwhile, for the Weibull fitting of the mean wind speed, the KS statistics and RMSE values obtained after bridge construction are generally higher than those prior to construction. However, as shown in Figure A1, the probability densities of the two different periods before bridge construction are highly similar, and the Kolmogorov–Smirnov (KS) test and root mean square error (RMSE) for the Weibull distribution fitting are both very small, with the coefficient of determination exceeding 0.96. This indicates that the bridge structure had an impact on the probability distribution of mean wind speed. Under natural conditions, the statistical characteristics of wind speed in the atmospheric boundary layer are typically well described by a single Weibull distribution. However, after the bridge is constructed, the bridge tower acts as bluff bodies, causing flow separation and generating wake regions on the leeward side, which leads to a noticeable velocity deficit. As a result, the disturbed wind speed reflects a mix of free-stream flow and wake-affected flow, which skews the probability distribution by increasing the weight in the lower wind speed range and, consequently, reduces the goodness-of-fit of the Weibull model. This reduction in goodness-of-fit is limited and the Weibull distribution still provides a satisfactory description of the probability density of mean wind speed.

To quantify the variations in average wind speed induced by the bridge, the Kolmogorov–Smirnov (KS) test was conducted. Due to the large sample size, one-fifth of the data was randomly sampled, and the KS test was repeated 1000 times, with the average result reported. This approach was applied consistently in all analyses. The statistical results, including *p*-values and KS statistics (*D*), are summarized in Table 1. When the measurement height is below the bridge tower (H < 142 m), the *p*-values are consistently below 0.05. The relatively large *D* values further confirm that the observed differences are not only statistically significant, but also physically substantial. In contrast, once the height exceeds 142 m, the *p*-values increase above 0.05 and the *D* statistics drop below 0.1, suggesting a negligible discrepancy between the two datasets. Furthermore, as a baseline comparison, the significance tests for the pre-construction period yield *p*-values consistently exceeding 0.05 and *D* values below 0.1, which demonstrate minimal inter-annual variability across the corresponding months prior to construction. Collectively, these findings indicated that the aerodynamic interference of the bridge tower on wind speed distributions is primarily confined within its vertical structural extent.

The 10 min mean wind speed at various heights before and after the bridge’s completion, along with their maximum values, are recorded in Table 2. It is evident that wind speed at all heights are generally lower after the bridge’s construction. However, as height increases, the difference between pre-construction and post-construction 10 min mean wind speed gradually decreases, stabilizing markedly above 142 m, corresponds to the height of the bridge tower’s top. However, before the bridge construction, the differences in the corresponding months of consecutive years within the bridge tower height range are all below 5.4% (see Table A1). At the same time, the maximum 10 min mean wind speed show larger discrepancies between pre-construction and post-construction conditions at lower elevations. In contrast, the differences in the maximum 10 min mean wind speed for the corresponding months of consecutive years before the bridge construction are all below 10%. The wind field at higher elevations experience minimal interference from bridge tower structures, although wind speed differences still persist. Moreover, slight differences in the mean wind speed values are also observed before the bridge construction. However, these differences can be considered independent of structural effects and are more likely attributable to natural variability caused by climatic factors such as atmospheric circulation. As a result, the greater disparity in the wind field observed at lower elevations before and after the bridge’s construction is attributed to interference from the bridge tower, which reduces wind speed. Underestimating wind speed leads to a non-linear reduction in calculated wind loads ($F\propto U^{2}$), potentially resulting in insufficient structural safety margins. Such inaccuracies risk structural damage during extreme wind events, leading to increased maintenance costs and compromised operational safety. Based on the above findings, wind speed within the height range influenced by the bridge tower (i.e., below 142 m) should be corrected in wind-resistant design to avoid underestimation of both the mean wind speed and the maximum 10 min mean wind speed. In contrast, above the tower height, attention should be paid to wind speed recovery, which may result in increased mean wind loads and amplified fluctuating wind effects due to the re-establishment of vertical wind speed gradients.

**Figure 5:**
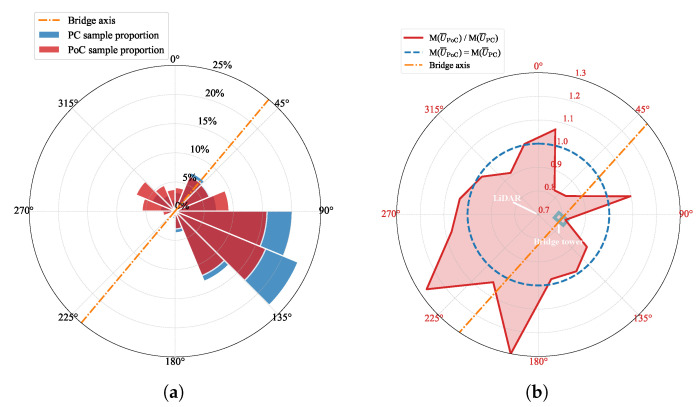
Wind rose and directional mean wind speed ratio (PoC/PC) at the 56 m height measurement point. M() denotes the average value within each wind direction sector. (**a**) Wind direction frequency distribution. (**b**) Directional mean wind speed ratio, $M\left({\bar{U}}_{\mathrm{PoC}}\right)/M\left({\bar{U}}_{\mathrm{PC}}\right)$.

The probability density function of the 10 min mean wind speed is affected by the presence of bridge structures. However, since the LiDAR is positioned on one side of the bridge structure, the measured wind speed from different directions may exhibit variations due to structural interference. To investigate these variations, a wind rose and a radar diagram are plotted as shown in Figure 5. The same wind direction sector division method is adopted, and the mean wind speed within each sector is averaged for comparison. After bridge construction, the proportion of wind direction in sectors $\left[90^{\circ },180^{\circ }\right]$ decreased, while the occurrence of wind direction in sectors $\left[270^{\circ },360^{\circ }\right]$ increased. At the same time, the mean wind speed associated with the wind directions observed most frequently in sectors $\left[90^{\circ },180^{\circ }\right]$ decreases after the bridge is built. This change is consistent with the layout of the site: the LiDAR is located on the northwest side of the bridge, and the wind that originated from the sectors $\left[90^{\circ },180^{\circ }\right]$ is weakened by the structural shielding of the bridge. It is clearly evident that the wind passing through the bridge tower experiences the most significant reduction. In other directions, the sample size is relatively small and the variations in wind speed before and after the bridge’s construction display a degree of randomness.

**Figure 6:**
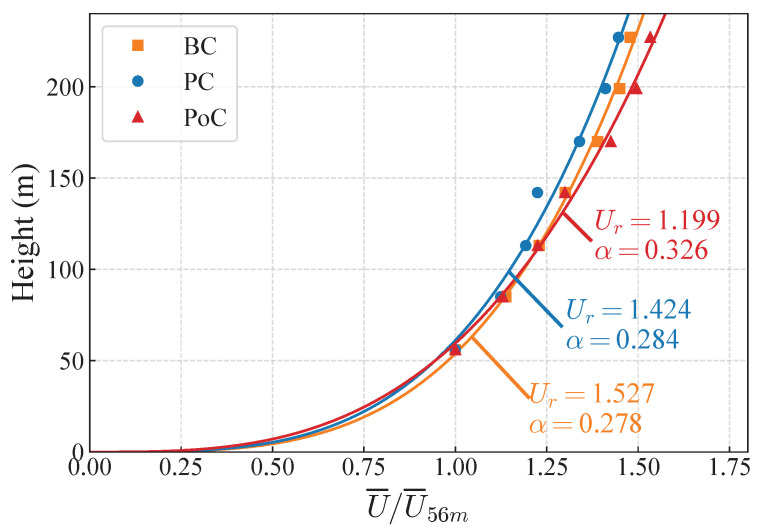
Ten-minute mean wind speed profiles during the BC, PC, and PoC periods.

The full sample of 10 min mean wind speed was selected to investigate the influence of the bridge tower on the vertical growth pattern of the mean wind speed. Exponential fitting was applied to the full sample, and the resulting wind speed profile is shown in Figure 6. The parameters of the exponential law for the 10 min mean wind speed at a height of 10 m were determined by the least squares method. The equation is expressed as follows [43]:

(13) $$U_{z}=U_{r}{\left(\frac{Z}{10}\right)}^{\alpha }$$

where $U_{z}$ represents the mean 10 min average wind speed at height *z*, $U_{r}$ denotes the mean 10 min average wind speed at 10 m, and α is the wind profile exponent. The wind profiles during the pre-construction period (PC) and the baseline period (BC) showed negligible differences, with the surface roughness coefficients differing by about 2%. The wind profile exponent is greater due to the influence of the bridge structure, with a difference of approximately 15%. This result suggests that the vertical wind speed gradient increases, with wind speed rising more rapidly with height after the bridge is constructed. The presence of the bridge structure impedes near-surface wind flow. The attenuated vertical gradient distorts the standard wind profile, leading to miscalculated load distributions across the tower height. This modification also alters the spatial correlation and aerodynamic damping, which are critical for the bridge’s aeroelastic stability and fatigue assessment.

### 3.2. Wind Characteristics Along the Bridge Deck Axis

**Figure 7:**
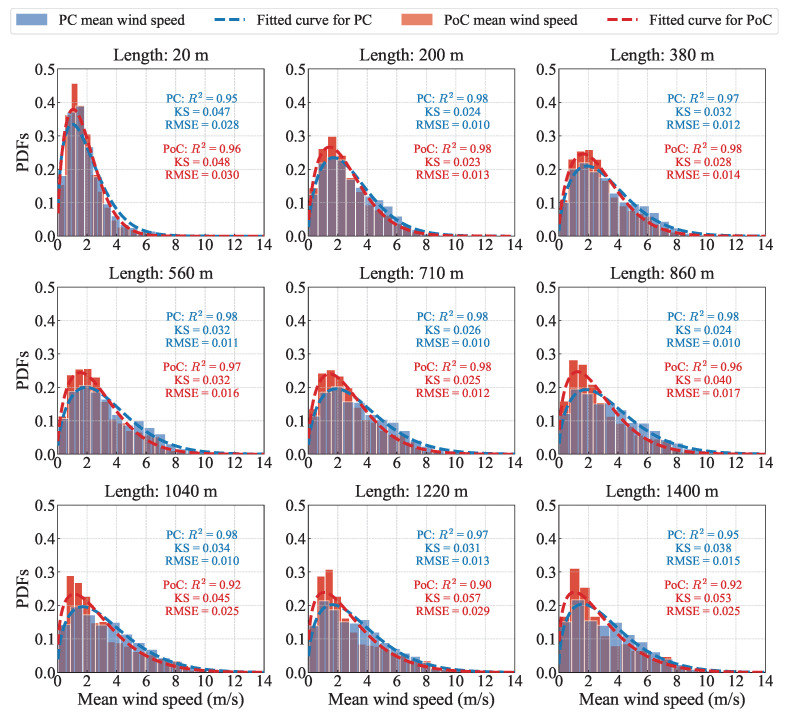
Probability density comparison of 10 min mean wind speed between PC and PoC periods at different measurement points along the bridge deck direction.

**Figure A2:**
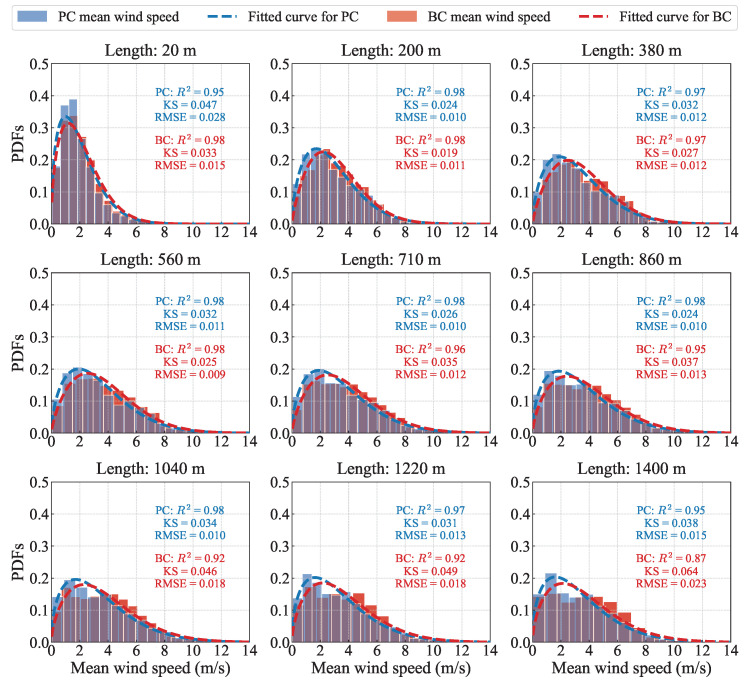
Probability density comparison of 10 min mean wind speed between BC and PC periods before bridge construction at different measurement points along the bridge deck.

To investigate the statistical characteristics of the 10 min mean wind speed measured along the bridge deck, a probability density curve was plotted, as shown in Figure 7. Similarly to the description in Section 3.1, the probability density curve after bridge construction becomes more concentrated, with the peak corresponding to a lower wind speed. Before bridge construction, the probability density shapes for the same months across two consecutive years are largely consistent. For the mean wind speed, we fit the data to a Weibull distribution. It is evident that, when taking the midpoint of the span as a reference, the coefficients of determination for the mean wind speed at measurement points closer to the LiDAR are nearly identical before and after the bridge’s construction. However, at measurement points beyond the midpoint and farther from the LiDAR, $R_{\mathrm{PoC}}^{2}$ are significantly lower than $R_{\mathrm{PC}}^{2}$, with values even dropping to a minimum of 0.9. Similar patterns are observed in the reference group (see in Figure A2), likely due to poorer observational quality at distant points. Meanwhile, when the Weibull distribution is used to fit the mean wind speed, the RMSE and KS statistics showed little change between pre- and post-construction periods. Considering all three indicators comprehensively, it can be concluded that the presence of the bridge deck impacted the probability distribution of mean wind speed. Nevertheless, the Weibull distribution still demonstrates good applicability in describing the statistical characteristics of the mean wind speed.

To evaluate the influence of the bridge girder structure on the spanwise wind field, the Kolmogorov–Smirnov (KS) test was conducted. The statistical results, including *p*-values and KS statistics (*D*), are summarized in Table 3. For the reference group, except at the 1400 m position, the *p*-values are all greater than 0.05, and the test statistics are relatively small, around 0.11, indicating that the probability distribution of the mean wind speed shows no significant differences. However, in the comparison before and after bridge construction, at nearly all measurement positions (from 380 m to 1400 m), the *p*-values are less than 0.05, demonstrating a statistically significant difference in the mean wind speed before and after construction. Notably, the test statistic reached a maximum of 0.187 at the mid-span region, suggesting that the main girder structure has the strongest regulating effect on the wind field at the mid-span.

Table 4 summarizes the statistical characteristics of wind speed. After the bridge is constructed, both the mean and maximum wind speed decrease, with the reduction in mean wind speed being most pronounced at the mid-span, reaching 22.35%. In contrast, for the reference group before bridge construction, the differences in mean wind speed across the measurement positions remain around 10% (see in Table A2), primarily due to natural variations caused by atmospheric circulation and other climatic factors. The increase in the difference of mean wind speed toward the mid-span after construction indicates that the flow environment is more affected by bridge construction in the mid-span region. In contrast, the measurement points near the bridge tower and the side span are already influenced by terrain-induced disturbances before construction, resulting in a comparatively smaller incremental impact after the bridge is completed. Furthermore, the along-bridge wind field should be appropriately corrected, especially in the mid-span region where the interference effects of the bridge deck and tower are most significant, to avoid errors in the estimation of aerodynamic loads.

**Figure 8:**
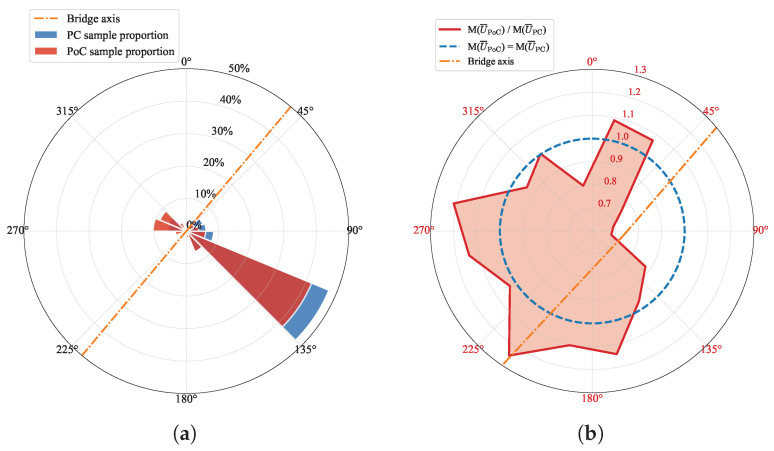
Wind rose and directional mean wind speed ratio (PoC/PC) at the mid-span measurement point. M() denotes the average value within each wind direction sector. (**a**) Wind direction frequency distribution. (**b**) Directional mean wind speed ratio, $M\left({\bar{U}}_{\mathrm{PoC}}\right)/M\left({\bar{U}}_{\mathrm{PC}}\right)$.

Similarly, to investigate the impact of the bridge structure on the wind speed in different wind directions, the wind speed direction rose and the ratio factor radar diagram were plotted, as shown in Figure 8. Figure 8 shows that the wind speed samples at the mid-span measurement point largely maintain consistent flow directions before and after bridge construction. A comparison of the wind direction frequencies in Figure 5a and Figure 8a indicates that the dominant wind sectors did not undergo significant shifts. Constrained by the valley terrain, the wind tends to follow the orientation of the valley, roughly perpendicular to the bridge’s axis. Therefore, the wind directions are predominantly in sectors $\left[90^{\circ },180^{\circ }\right]$, with the majority of directions falling in sectors $\left[112.5^{\circ },135^{\circ }\right]$. Wind speed originating from the east experience a noticeable decrease after the bridge is completed. This change aligns with the description in Section 3.1, suggesting that the reduction in wind speed in this direction is due to structural interference caused by the bridge deck.

## 4. Effect of Bridge Tower on Turbulence Wind Characteristics

### 4.1. Turbulence Intensity

**Figure 9:**
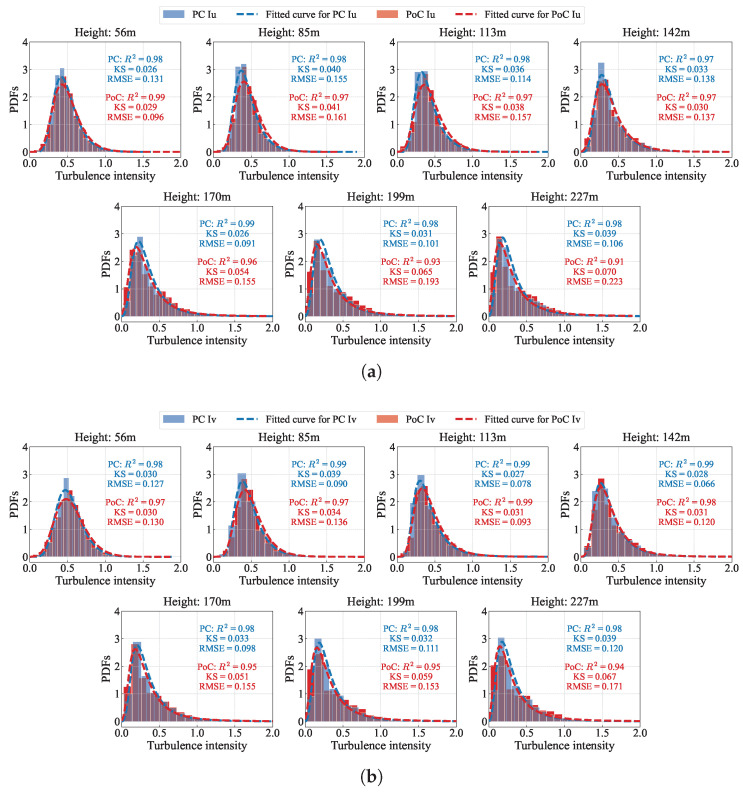
Probability density comparison of turbulence density between PC and PoC periods at different measurement points along the bridge tower direction. (**a**) $I_{u}$; (**b**) $I_{v}$.

Turbulence intensity characterizes the fluctuations in wind speed and the degree of turbulent motion within the atmospheric boundary layer. It plays a crucial role in determining the impact of wind loads on structures. To investigate the effect of structural interference on the turbulence intensity distribution, a comparative analysis of the turbulence intensity probability densities before and after the bridge construction was conducted, as shown in Figure 9. It can be observed that, whether for longitudinal or lateral turbulence intensity, the probability density distributions before and after the bridge construction are similar, with slight differences in shape. In most cases, the probability density function after construction has a lower peak. Moreover, for heights below 142 m, the peak of post-construction corresponds to a higher turbulence intensity, while the opposite trend is observed above 142 m.

For turbulence intensity, we fit the corrected data to a Generalized Extreme Value (GEV) distribution. The GEV distribution is well-suited for modeling turbulence intensity due to its ability to capture the asymmetric and bounded characteristics of turbulence data, which frequently show extreme value behavior in atmospheric boundary-layer flows [44,45]. The probability density functions (PDFs) of the GEV distribution is expressed as follows:

(14) $$f\left(s;\xi \right)=\left\{\begin{array}{cc} \exp\left(-s\right)\exp\left[-\exp\left(-s\right)\right] & \mathrm{if}\;\xi =0,\\ {\left(1-\xi s\right)}^{1/\xi -1}\exp\left[-{\left(1-\xi s\right)}^{1/\xi }\right] & \mathrm{if}\;\xi \neq 0\;\mathrm{and}\;\xi s<1,\\ 0 & \mathrm{otherwise}, \end{array}\right.$$

where $s=\frac{x-\mu }{\sigma }$, μ is the location parameter, σ is the scale parameter, and ξ is the shape parameter.

**Figure A3:**
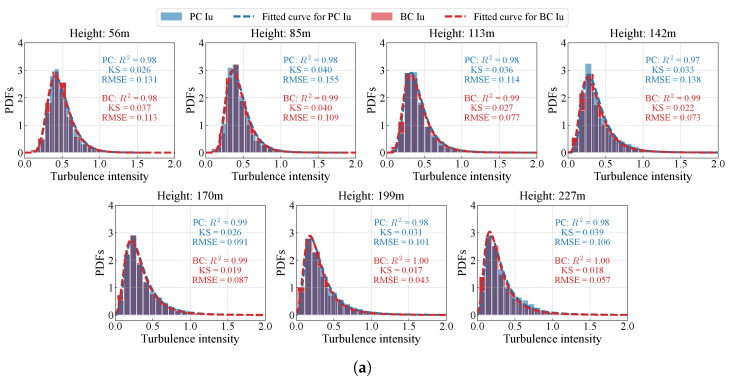

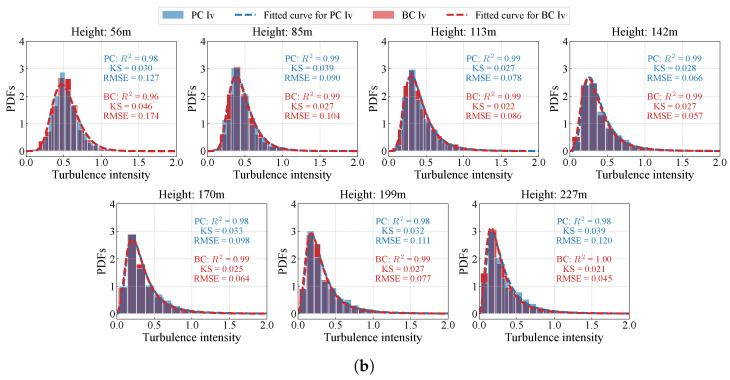
Probability density comparison of turbulence density between BC and PC periods at different measurement points along the bridge tower direction. (**a**) $I_{u}$; (**b**) $I_{v}$.

Figure 9 present the comparative probability density functions of the turbulence density under pre-construction and post-construction. After the bridge was constructed, the $R_{\mathrm{PoC}}^{2}$ at various heights using the GEV distribution is almost lower than the $R_{\mathrm{PC}}^{2}$. Meanwhile, the KS statistics and RMSE values obtained after bridge construction are mostly higher than those prior to construction. However, as shown in Figure A3, the probability densities of both $I_{u}$ and $I_{v}$ during the two different periods before bridge construction are highly similar, and the KS test and RMSE for the GEV distribution fitting are both very small, with the coefficient of determination is generally above 0.96, reaching 0.99 or higher in some cases. This indicates that, after bridge construction, structure-induced turbulence introduces additional high-frequency and partially periodic fluctuations. The superposition of these coherent vortical structures with background turbulence weakens the independence assumption, leading to deviations in the tail fitting of the extreme value distribution. As a result, the goodness-of-fit of the GEV model decreases slightly, although it still maintains good descriptive capability.

To evaluate the influence of the bridge tower structure on the distribution of turbulence intensity, the Kolmogorov–Smirnov (KS) test is applied without imposing any wind speed threshold for data filtering. Statistical analyses are performed based on the *p*-value and the KS statistic (*D*). The results are summarized in Table 5. The statistical results indicate that, except around the height of 142 m (corresponding to the top of the bridge tower), the *p*-values are smaller than 0.05, suggesting statistically significant differences between the pre-construction and post-construction periods. However, the corresponding *D* values remain relatively small (|D|<0.11) at all heights, indicating that, although statistically detectable differences exist, the practical magnitude of these differences remains limited.

**Figure A4:**
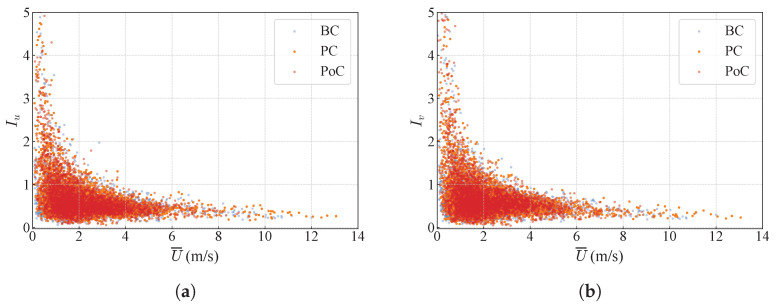
Variation in turbulence intensity with mean wind speed during BC, PC and PoC periods. (**a**) Longitudinal turbulence intensity ($I_{u}$). (**b**) Lateral turbulence intensity ($I_{v}$).

Turbulence intensity values at lower wind speeds are not particularly representative. As shown in Figure A4, with the increase of wind speed, the turbulence intensity gradually approaches a stable value. When the mean wind speed exceeds 7 m/s, the turbulence intensity becomes relatively stable, and the calculated turbulence intensity is therefore more representative. Consequently, a data filtering threshold of a mean wind speed of 7 m/s is applied.Table 6 and Table 7 present a comparison of longitudinal turbulence intensity ($I_{u}$) and lateral turbulence intensity ($I_{v}$) under pre-construction and post-construction conditions. A comparison of turbulence intensity during the corresponding months of consecutive years before bridge construction (BC and PC) shows that the differences at various heights are irregular, with longitudinal turbulence intensity differences below 7% and transverse turbulence intensity differences below 11%. When comparing turbulence intensity before and after bridge construction, it is noteworthy that the height threshold remains at 142 m, which corresponds to the height of the bridge tower’s top. Below this height, post-construction yields a higher turbulence intensity, while above this height, the turbulence intensity is lower, with differences exceeding 30%. This phenomenon can be explained as follows: below the tower height, the structure acts as a bluff body, triggering strong flow separation and a highly turbulent wake. This creates a mean velocity deficit, while vortex shedding amplifies high-frequency turbulent energy in the power spectrum. Consequently, the reduced mean wind speed combined with increased velocity fluctuations sharply elevates local turbulence intensity. Conversely, above the tower, the airflow escapes the structural wake and experiences an aerodynamic speed-up effect. Furthermore, the tower’s physical blockage disrupts large-scale atmospheric eddies, effectively attenuating low-frequency spectral energy. This combination of an accelerated mean wind speed and reduced total turbulent kinetic energy significantly lowers turbulence intensity, occasionally even below pre-construction baselines. This localized redistribution of turbulence intensity can lead to an underestimation of gust loads on lower structural components while causing inaccurate fatigue and vibration assessments due to the deviation from standard atmospheric turbulence models. Therefore, the interference effect of the bridge tower structure on turbulence intensity below the tower height should be considered, and appropriate correction of turbulence intensity within the tower-influenced zone is necessary for accurate wind-resistant design.

**Figure 10:**
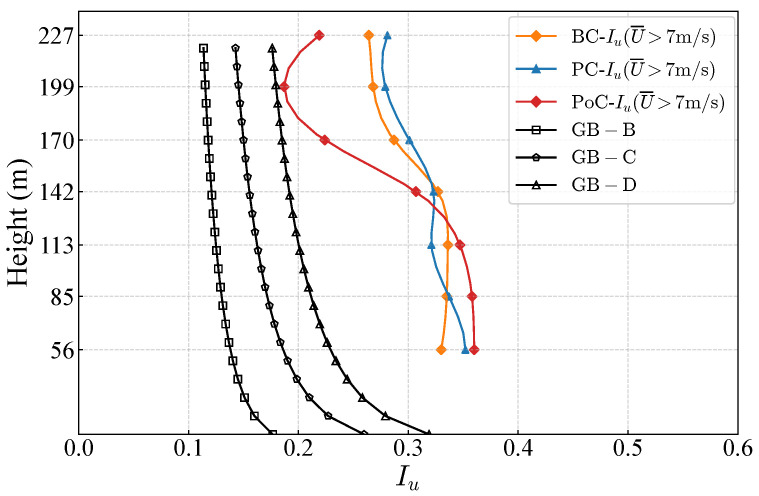
Longitudinal turbulence density profile during BC, PC, and PoC periods.

To provide a more intuitive analysis of how turbulence intensity varies with height, turbulence intensity profiles were plotted, as shown in Figure 10. It can be observed that, compared to the longitudinal turbulence intensity values specified in the Chinese National Standards, the measured values are consistently higher. This discrepancy can likely be attributed to the strong disturbances induced by the mountainous valley terrain, which cause significant airflow variations and flow separation, thereby amplifying turbulence intensity. Before bridge construction, the differences in $I_{u}$ between corresponding months of consecutive years are relatively small. However, after the bridge was constructed, the turbulence intensity profile under high wind speed conditions exhibits a pronounced S-shaped pattern, significantly deviating from the expected exponential distribution. The inflection point is approximately located at the height of the bridge tower top, further indicating that the bridge tower structure interferes with the turbulence intensity profile under strong wind conditions.

**Figure 11:**
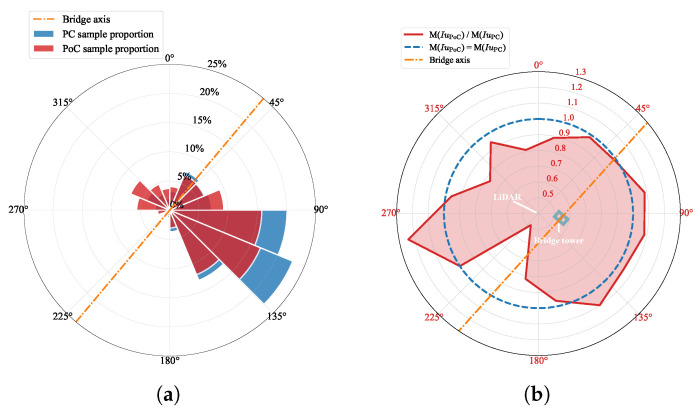
Wind rose and directional turbulence intensity ratio (PoC/PC) at the 56 m measurement point. M() denotes the average value within each wind direction sector. (**a**) Wind direction frequency distribution. (**b**) Directional mean turbulence intensity ratio, $M\left({I_{u}}_{\mathrm{PoC}}\right)/M\left({I_{u}}_{\mathrm{PC}}\right)$.

To further investigate the turbulence characteristics in the vicinity of the bridge tower, turbulence intensity was analyzed in relation to the prevailing wind direction using rose diagrams, as shown in Figure 11. The sample size for wind directions in sectors $\left[180^{\circ },270^{\circ }\right]$ is too small to be considered reliable. In all other directions, after the bridge was completed, the LiDAR observed higher turbulence intensity coming from the direction of the bridge tower, while the opposite trend occurred in the remaining directions. It is not difficult to infer that, in the absence of the bridge structure, the turbulence intensity observed in recent measurements would decrease, whereas, because of the influence of the bridge tower structure, the disturbed turbulence intensity would increase. This further confirms that the bridge tower structure causes interference in wind field measurements.

### 4.2. Power Spectrum Density

The fluctuating wind power spectrum is a parameter that characterizes the distribution of turbulent wind energy in the frequency domain. According to much current research on field measurement, the von Kármán spectrum is one of the most suitable wind spectra to represent the fluctuating wind power spectrum in mountain valleys terrains [5,6]. The expression for the von Kármán spectrum is as follows:

(15) $$\frac{f\cdot S_{u}\left(f,z\right)}{\sigma_{u}^{2}}=\frac{\frac{4L_{u}\cdot f}{\bar{U}}}{{\left[1+70.8{\left(\frac{L_{u}\cdot f}{\bar{U}}\right)}^{2}\right]}^{5/6}}$$

where *f* represents the turbulent frequency of the wind; $S_{u}\left(f,z\right)$ is the longitudinal turbulent wind power spectrum at height *z*; $L_{u}$ is the longitudinal turbulent integral length scale; and $\bar{U}$ is the mean wind speed.

**Figure 12:**
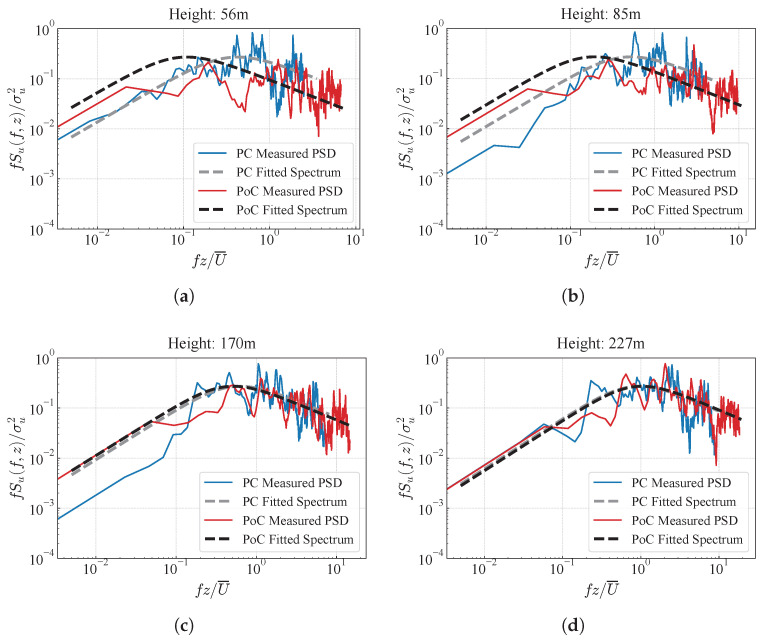
Fluctuating wind power spectra comparison between PC and PoC periods at different measurement points along the bridge tower direction. (**a**) 56 m; (**b**) 85 m; (**c**) 170 m; (**d**) 227 m.

**Figure 13:**
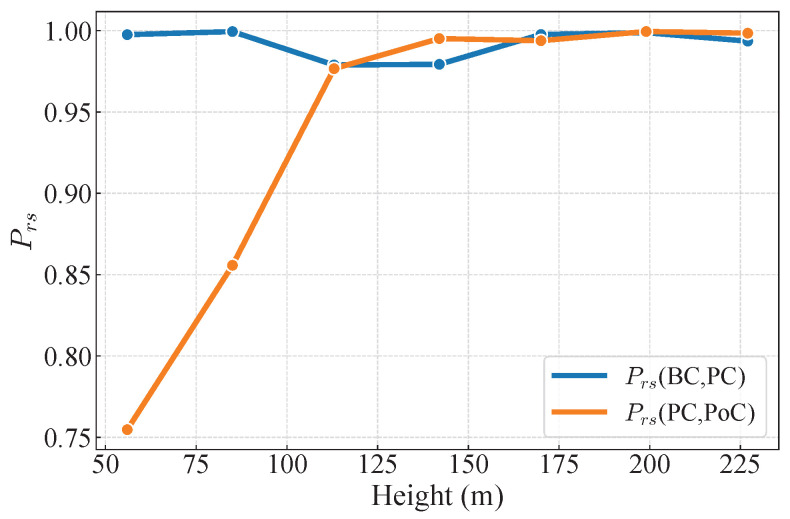
The Pearson correlation coefficient $P_{rs}$ between fitted power spectra among BC, PC, and PoC periods.

A comparative analysis is conducted by fitting the fluctuating longitudinal wind power spectrum before and after bridge construction to the von Kármán spectrum, as shown in Figure 12. The similarity between the two power spectra is quantified using the Pearson correlation coefficient ($P_{rs}$), as shown in Figure 13, and the formula for $P_{rs}$ is as follows [46]:

(16) $$\rho_{S_{1},S_{2}}=\frac{E\left[\left(S_{1}-E\left[S_{1}\right]\right)\left(S_{2}-E\left[S_{2}\right]\right)\right]}{\sigma_{S_{1}}\sigma_{S_{2}}}$$

where $S_{1}$ and $S_{2}$ are the energy calculated from the fitting formula by von Kármán spectrum before and after the construction of the bridge, respectively. From Figure 13, it can be observed that the differences between the power spectra corresponding to the same months in consecutive years before bridge construction are very small. Therefore, the differences between the power spectra before and after bridge construction can be considered to be caused by the presence of the bridge structure. As shown in Figure 12, it is evident that, due to the interference caused by the bridge tower, the measured fluctuating wind power spectrum exhibits a lower degree of fit with the von Kármán spectrum, particularly at low-frequency components, which are below the von Kármán spectrum. As the height increases, the degree of fitting improves, indicating that the interference of the bridge tower on the turbulent wind characteristics decreases. Moreover, as height increases, the discrepancy between the fluctuating wind power spectra from different periods gradually decreases, a trend that is clearly visible in Figure 13. Below the height of the bridge tower, the power spectra exhibit a noticeable difference, while above 125 m, the $P_{rs}$ approaches 1. This suggests that the presence of the bridge tower influences the distribution of turbulent energy. This effect is specifically reflected in the decrease in energy at moderate frequencies ($10^{-1}<fz/\bar{U}<10^{0}$) and the significant increase in high-frequency components ($fz/\bar{U}>10^{0}$) in the measured fluctuating wind power spectrum. This can be explained as follows: flow separation and vortex disturbances induced by the bridge tower structure suppress the energy in the moderate-frequency range, while local vortex and structural vibrations contribute to the increased energy in the high-frequency components.

## 5. Concluding Remarks

To investigate the influence of the bridge structure on the wind field at the bridge site in a mountain valleys terrain, field measurements were conducted using a laser wind LiDAR. The mean wind speed and turbulent characteristics before and after bridge construction were analyzed and compared, providing a valuable reference for correcting wind field measurements affected by the bridge. This study primarily reveals the impact of the bridge tower structure and the bridge deck structure on the wind field.

The bridge tower primarily affects the vertical wind field characteristics, including the mean wind speed, turbulence intensity, and fluctuating wind power spectrum. The main conclusions are as follows:

1. The presence of the bridge tower reduces the degree of fit between the probability density of the mean wind speed and the Weibull distribution. Due to the interference of the bridge tower, the mean wind speed is reduced by approximately 7%–10% within the tower height range and by about 4% outside this range, particularly for wind coming from the direction of the bridge tower, as observed by the LiDAR. Additionally, the bridge tower slightly weakens the vertical increase in wind speed. Therefore, engineering assessments should correct the wind speed within the tower-influenced region.
2. The turbulence intensity shows a poorer fit to the GEV distribution due to the influence of the bridge tower. Within the height range of the bridge tower, turbulence intensity increases by approximately 1%–10%, while it decreases by about 20%–33% in regions above the top of the tower. Engineering projects should account for the interference-induced amplification of measured turbulence intensity within the height of bridge tower to prevent overestimation of structural safety.
3. The turbulence wind power spectrum affected by the bridge tower cannot be accurately described by the VK spectrum, with a reduction in energy at medium frequencies and an increase in high-frequency components.

The bridge deck primarily affects the along-bridge wind field characteristics, including the mean wind speed and its probability distribution. The main conclusions are as follows:

1. The presence of the bridge deck affects the probability distribution of the mean wind speed, but the Weibull distribution remains highly applicable.
2. The wind speed on the side of the bridge deck is generally reduced, with a reduction of approximately 10%–22%. Therefore, in engineering projects, it is important to consider the potential risk of reduced wind speed measurements, which may require adjustments to structural design parameters and assessments of driving safety.

Unfortunately, the LiDAR has only one measurement line along the direction of the bridge tower and one along the bridge deck, making it impossible to perform synchronized observational validation. To improve this, more measurement lines should be set up to obtain more comprehensive data, allowing correction of measurement errors.

## Figures and Tables

**Table 1:** KS test results for wind speed distributions at different measurement points along the bridge tower. Left: Post- vs. pre-construction (PC-PoC). Right: Baseline comparison before bridge construction (BC-PC).

Height (m)	PC-PoC		Baseline Comparison	
	*p*-Value	*D*	*p*-Value	*D*
56	2.16 $\times \;10^{-3}$	0.118	3.82 $\times \;10^{-1}$	0.049
85	1.52 $\times \;10^{-3}$	0.121	2.59 $\times \;10^{-1}$	0.055
113	2.39 $\times \;10^{-2}$	0.095	9.91 $\times \;10^{-2}$	0.073
142	1.31 $\times \;10^{-1}$	0.069	1.72 $\times \;10^{-2}$	0.093
170	1.39 $\times \;10^{-1}$	0.068	5.73 $\times \;10^{-2}$	0.080
199	8.00 $\times \;10^{-2}$	0.076	1.13 $\times \;10^{-1}$	0.070
227	6.72 $\times \;10^{-2}$	0.079	1.46 $\times \;10^{-1}$	0.064

**Table 2:** Mean wind speed statistics comparison between PC and PoC periods at different measurement points along the bridge tower. Δ is variation ratio.

Height (m)	Mean (m/s)			Max (m/s)		
	PC	PoC	Δ (%) *	PC	PoC	Δ (%)
56	2.38	2.14	−10.08	13.06	9.29	−28.87
85	2.67	2.42	−9.36	12.85	9.42	−26.69
113	2.84	2.63	−7.39	12.86	9.38	−27.06
142	2.91	2.79	−4.12	12.54	10.00	−20.26
170	3.19	3.05	−4.39	13.32	10.33	−22.45
199	3.36	3.21	−4.46	14.10	10.46	−25.82
227	3.44	3.29	−4.36	14.54	11.30	−22.28

* $\Delta =\frac{Value(\mathrm{PoC})-Value(\mathrm{PC})}{Value(\mathrm{PC})}\times 100\%$.

**Table 3:** KS test results for wind speed distributions at different measurement points along the bridge deck. Left: Post-construction vs. pre-construction (PC-PoC). Right: Baseline comparison before bridge construction (BC-PC).

Lenght (m)	PC-PoC		Baseline Comparison	
	*p*-Value	*D*	*p*-Value	*D*
20	2.24 $\times \;10^{-1}$	0.085	1.70 $\times \;10^{-1}$	0.094
200	5.90 $\times \;10^{-2}$	0.115	7.60 $\times \;10^{-2}$	0.111
380	2.70 $\times \;10^{-2}$	0.128	5.57 $\times \;10^{-2}$	0.117
560	5.66 $\times \;10^{-3}$	0.154	5.61 $\times \;10^{-2}$	0.118
710	4.44 $\times \;10^{-3}$	0.158	8.09 $\times \;10^{-2}$	0.111
860	4.86 $\times \;10^{-4}$	0.187	6.94 $\times \;10^{-2}$	0.114
1040	3.95 $\times \;10^{-3}$	0.154	6.57 $\times \;10^{-2}$	0.113
1220	4.11 $\times \;10^{-3}$	0.161	6.87 $\times \;10^{-2}$	0.113
1400	4.25 $\times \;10^{-3}$	0.160	3.87 $\times \;10^{-2}$	0.124

**Table 4:** Mean wind speed statistics comparison between PC and PoC periods at different measurement points along the bridge deck.

Lenght (m)	Mean (m/s)			Max (m/s)		
	PC	PoC	Δ (%)	PC	PoC	Δ (%)
20	1.99	1.79	−10.05	12.07	8.95	−25.85
200	2.90	2.54	−12.41	12.73	12.70	−0.24
380	3.24	2.77	−14.51	13.60	11.50	−15.44
560	3.39	2.76	−18.58	14.62	11.50	−21.34
710	3.45	2.79	−19.13	15.35	12.00	−21.82
860	3.49	2.71	−22.35	15.30	12.70	−16.99
1040	3.43	2.88	−16.03	16.90	11.10	−34.32
1220	3.32	2.82	−15.06	13.80	11.10	−19.57
1400	3.30	2.82	−14.55	17.20	13.90	−19.19

**Table 5:** KS test results for turbulence intensity distributions between PC and PoC periods at different measurement points along the bridge tower.

Height (m)	$I_{u}$		$I_{v}$	
	*p*-Value	*D*	*p*-Value	*D*
56	2.70 $\times \;10^{-2}$	−0.089	1.57 $\times \;10^{-2}$	−0.095
85	1.08 $\times \;10^{-2}$	−0.102	2.90 $\times \;10^{-2}$	−0.090
113	3.92 $\times \;10^{-2}$	−0.086	1.14 $\times \;10^{-1}$	−0.071
142	2.50 $\times \;10^{-1}$	−0.056	3.82 $\times \;10^{-1}$	−0.050
170	2.54 $\times \;10^{-2}$	−0.082	7.58 $\times \;10^{-2}$	−0.071
199	2.04 $\times \;10^{-2}$	−0.084	3.41 $\times \;10^{-2}$	−0.078
227	1.14 $\times \;10^{-2}$	−0.090	2.36 $\times \;10^{-2}$	−0.083

**Table 6:** Longitudinal turbulence intensity value comparison at different measurement points along the bridge tower direction.

Height (m)	PC	BC	Δ _*base*_ * (%)	PoC	Δ (%)
56	0.343	0.331	6.99	0.365	2.27
85	0.354	0.370	0.60	0.389	6.23
113	0.343	0.384	−4.46	0.366	8.10
142	0.337	0.367	−1.22	0.340	−5.25
170	0.316	0.322	4.88	0.232	−25.83
199	0.286	0.297	4.10	0.193	−32.97
227	0.276	0.284	6.44	0.221	−22.06

* $\Delta_{base}=\frac{Value(\mathrm{PC})-Value(\mathrm{BC})}{Value(\mathrm{BC})}\times 100\%$.

**Table 7:** Lateral turbulence intensity value comparison at different measurement points along the bridge tower direction.

Height (m)	PC	BC	Δ _*base*_ (%)	PoC	Δ (%)
56	0.344	0.331	3.93	0.365	6.1
85	0.354	0.370	−4.32	0.389	9.89
113	0.344	0.384	−10.42	0.366	6.40
142	0.337	0.367	−8.17	0.340	−0.89
170	0.317	0.322	−1.55	0.232	−26.81
199	0.286	0.298	−4.03	0.193	−32.52
227	0.276	0.284	−2.82	0.221	−19.93

## Data Availability

Data will be made available on request.

## Appendix A. Supplementary Analysis for Effect of Bridge Structure on Mean Wind Characteristics

**Table A1:** Mean wind speed statistics comparison between pre-construction (PC) and before-construction (BC) periods at different measurement points along the bridge tower.

Height (m)	Mean (m/s)			Max (m/s)		
	PC	BC	Δ _*base*_ (%) *	PC	BC	Δ _*base*_ (%)
56	2.38	2.44	−2.46	13.06	12.00	8.83
85	2.67	2.77	−3.61	12.85	11.77	9.17
113	2.84	3.00	−5.33	12.86	12.04	6.81
142	2.91	3.17	−8.20	12.54	12.08	3.81
170	3.19	3.39	−5.90	13.32	12.99	2.54
199	3.36	3.53	−4.82	14.10	13.46	4.75
227	3.44	3.60	−4.44	14.54	13.96	4.15

* $\Delta_{base}=\frac{Value(\mathrm{PC})-Value(\mathrm{BC})}{Value(\mathrm{BC})}\times 100\%$.

**Table A2:** Mean wind speed statistics comparison between pre-construction (PC) and before-construction (BC) periods at different measurement points along the bridge deck.

Lenght (m)	Mean (m/s)			Max (m/s)		
	PC	BC	Δ _*base*_ (%)	PC	BC	Δ _*base*_ (%)
20	1.99	2.14	−7.01	12.07	12.27	−1.63
200	2.90	3.16	−8.23	12.73	10.85	17.33
380	3.24	3.58	−9.50	13.60	11.90	14.29
560	3.39	3.77	−10.08	14.62	17.00	−14.00
710	3.45	3.84	−10.16	15.35	13.34	15.07
860	3.49	3.89	−10.28	15.30	13.90	10.07
1040	3.43	3.81	−9.97	16.90	13.50	25.19
1220	3.32	3.69	−10.03	13.80	17.10	−19.30
1400	3.30	3.72	−11.29	17.20	19.90	−13.57
